# Distribution of device-measured 24-hour movement behaviours across the cognitive spectrum in older adults: the HUNT study

**DOI:** 10.1186/s11556-026-00433-x

**Published:** 2026-08-13

**Authors:** Karen Sverdrup, Paul Jarle Mork, Eling D. de Bruin, Linda Ernstsen, Gill Livingston, Kushang V. Patel, Geir Selbæk, Bjørn Heine Strand, Beatrix Vereijken, Gro Gujord Tangen

**Affiliations:** 1 Norwegian National Centre for Ageing and Health, Sykehuset i Vestfold, Tønsberg, Norway; 2 Department of Geriatric Medicine, Oslo University Hospital, Oslo, Norway; 3 Department of Anesthesiology and Pain Medicine, University of Washington, Seattle, United States; 4 Department of Public Health and Nursing, Norwegian University of Science and Technology, Trondheim, Norway; 5 Motor Control and Learning Group - Institute of Human Movement Sciences and Sport, Department of Health Sciences and Technology, ETH Zurich, Zurich, Switzerland; 6 Department of Health, Ostschweizer Fachhochschule OST, Rapperswil, Switzerland; 7 Division of Psychiatry, University College London, London, United Kingdom; 8 North London partnership trust NHS Foundation Trust, St Pancreas Hospital, London, United Kingdom; 9 Division of Gerontology and Geriatric Medicine, Department of Medicine, University of Washington, Seattle, WA USA; 10 Department of Physical Health and Ageing, Norwegian Institute of Public Health, Oslo, Norway; 11 Department of Neuromedicine and Movement Science, Norwegian University of Science and Technology, Trondheim, Norway

**Keywords:** Device-measured, Neurodegenerative disorder, Body-worn sensor, Physical activity, Sedentary behaviour

## Abstract

**Background:**

Key daily physical activities (e.g., standing and walking), postures (sitting and lying), and sleep constitute the 24-h day and have distinct health effects and functional implications. Traditionally studied separately, the distribution of these movement behaviours and sleep across the cognitive spectrum remains insufficiently characterised. We aimed to describe and compare the distribution of 24-h movement behaviours and sleep across the cognitive spectrum.

**Methods:**

We analysed cross-sectional, population-based data from 4,699 community-dwelling older adults (≥ 70 years) participating in the HUNT4 70 + study. Cognitive status was classified as mild cognitive impairment (MCI, *n* = 1,690) or dementia (*n* = 360) according to DSM-5 criteria. Participants not classified with MCI or dementia were categorised as cognitively unimpaired. Accelerometers worn on the thigh and lower back (≤ 7 days) were used to assess standing, walking, sitting, lying awake, and sleep using validated machine-learning models. Inverse probability weighting and multiple imputation by chained equations were used to address selection bias. Group differences were examined using survey-weighted regression models adjusted for potential confounders including age, sex, education, marital status, self-rated health, obesity, depression, alcohol consumption, and smoking.

**Results:**

In fully adjusted models, older adults with MCI spent less time sitting (ß -12.4 [95% CI -20.0, -4.4] min/ 24 h) and more time lying awake (ß 10.3 [95% CI 2.7, 17.9]) and sleeping (ß 3.9 [95% CI 0.8, 7.0] min/24 h) than their cognitively unimpaired peers (all *p* < 0.05), while time spent standing and walking was similar between the two groups. In fully adjusted models, older adults with dementia spent less time standing (ß -29.0 [95% CI -41.3, -16.5] and ß -30.3 [95% CI -42.5, -18.2] min/24 h) and walking (ß -16.1 [95% CI -19.9, -12.4] and ß -17.2 [95% CI -20.9, -13.4] min/24 h), and more time lying awake (ß 36.3 [95% CI 17.1, 55.4] and ß 46.5 [95% CI 28.7, 64.5] min/24 h), than those with MCI and cognitively unimpaired peers (all p ≤ 0.001), and more time sleeping (ß 7.5 [95% CI 0.9, 14.9] min/24 h) than their cognitively unimpaired peers (*p* = 0.04).

**Conclusion:**

Across the cognitive spectrum, older adults exhibit distinct distributions of 24-h movement behaviours and sleep. The clinical importance of the observed differences remains unknown, and future research on the independent and compositional effects of these behaviours is warranted.

**Supplementary Information:**

The online version contains supplementary material available at https://doi.org/10.1186/s11556-026-00433-x.

## Background

In 2019, an estimated 57.4 million people were living with dementia worldwide, a figure projected to rise to 152.8 million by 2050 [1]. Mild cognitive impairment (MCI), an at-risk stage between normal cognitive function and dementia [2], affects about 15.6% of community-dwelling adults aged ≥ 50 years [3]. Physical activity (PA), sedentary behaviour (SB), and sleep are modifiable lifestyle factors associated with the risk of cognitive impairment and dementia [4–8]. Collectively, spending more time in PA, limiting SB, and ensuring adequate sleep duration is associated with better cognitive function in older adults and reduced risk of dementia [5–9]. Among individuals with MCI and dementia, spending more time in PA and limiting SB are also associated with maintenance of functioning in activities of daily living and physical performance [10–13]. However, previous studies show that people with dementia engage in less PA, more SB, and sleep more than their cognitively unimpaired peers [14–18], whereas findings in MCI are inconsistent [19–23]. Currently, it remains unclear whether discrete stages of cognitive impairment (i.e., MCI vs dementia) exhibit different PA, SB, and sleep distributions [14].

Advances in accelerometry and machine-learning classification models now further allow the untangling of PA and SB into distinct movement behaviours, including key daily physical activities (e.g., standing, walking, running, and cycling) and postures (sitting and lying) [24–26]. Together with sleep, they occupy most of the 24-h day in older adults [27]. Beyond distinct energy expenditure requirements, these key daily physical activities and postures have different functional implications and health effects [28–31]. Both standing and walking, which comprise the majority of everyday PA in older adults [27, 32], and sitting and lying, primary components of SB [33], reflect different levels of participation and behavioural and social context, and require distinct functional capacities. These movement behaviours and sleep are components of a finite 24-h day and inherently codependent [34]. Compositional data analysis has emerged as an important framework for examining the interdependencies among these behaviours and the implications of reallocating time between them [35]. However, before exploring the compositional relationships of these behaviours on cognitive health, there is a need to characterise differences in time accumulated in distinct behaviours across the cognitive spectrum. This may provide nuanced insights into PA and SB distributions in older adults, elucidate observed differences in PA and SB in dementia, and clarify inconsistencies in prior findings in MCI, thereby informing the development of targeted strategies to maintain function in older adults.

This population-based study aimed to describe and compare the distribution of 24-h movement behaviours and sleep across the cognitive spectrum in older adults. To achieve this, we leveraged one of the world's largest samples of older adults with a comprehensive, well-described cognitive assessment and diagnostic protocol [36, 37] and accelerometer-measured time spent in key daily physical activities, postures, and sleep [26, 38, 39].

## Methods

### Study design and participants

**Fig. 1:**
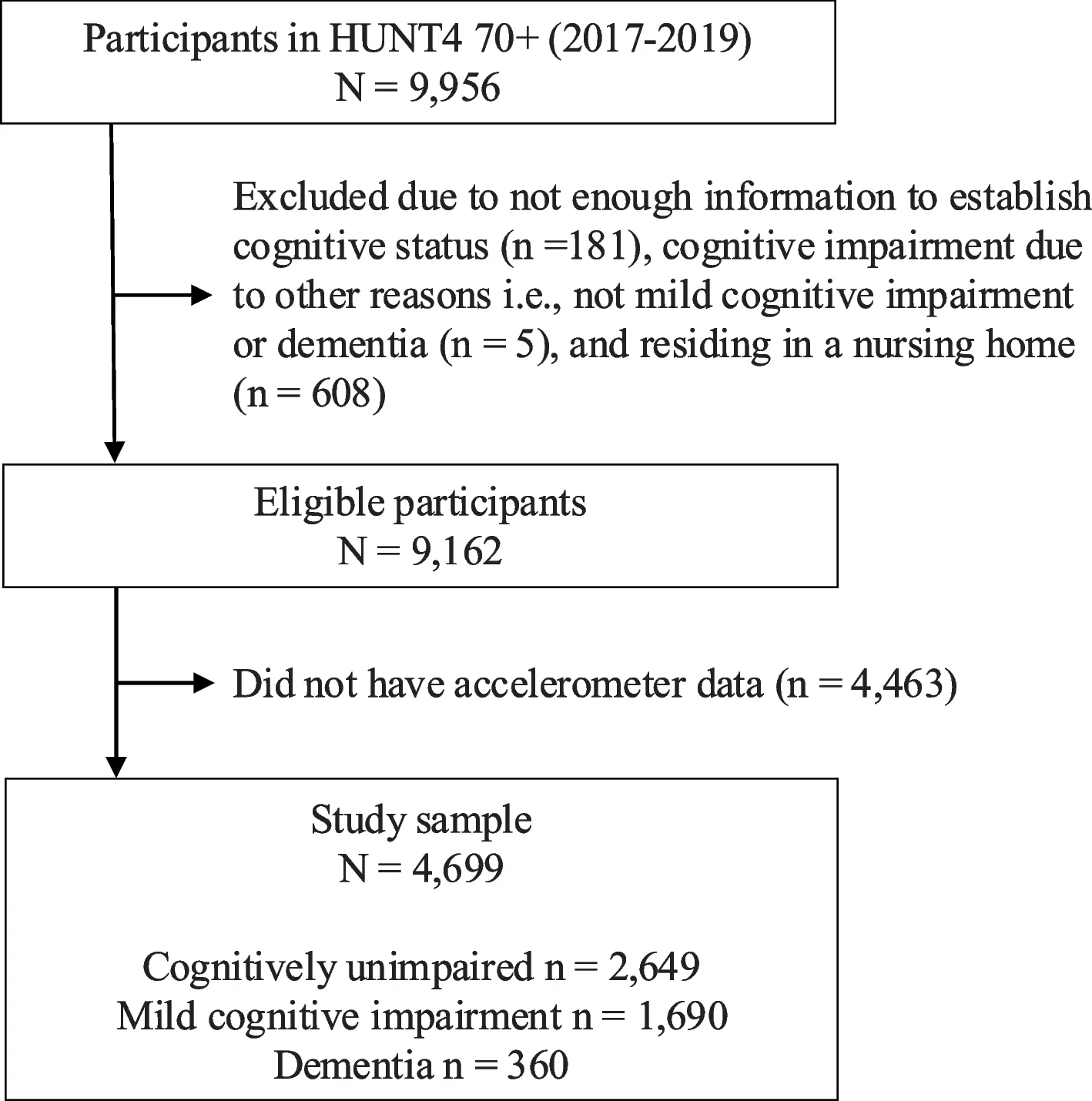
Flow-chart of included participants. HUNT4 70 + = The Trøndelag Health Study, 4th survey, 70 years-and-older cohort

This cross-sectional, population-based study used data from the 70-years-and-older cohort (*N* = 9,956) in the 4th survey of the Trøndelag Health Study (HUNT4 70 +) [40, 41]. Briefly, data collection took place between August 2017 and February 2019. Participants completed standardised questionnaires, and trained health professionals conducted clinical assessments of physical and cognitive function at designated field stations. Participants unable to attend field stations were offered assessments at their primary residence, including private homes and nursing homes. In the current study, participants were excluded if they lacked sufficient information to determine cognitive status (*n* = 181), had cognitive impairment(s) not attributable to MCI or dementia (*n* = 5), or were living in a nursing home (*n* = 608). Of the 9,162 eligible participants, 4,699 (51.3%) had valid accelerometer data (≥ 1 day) and were included in this study (Fig. 1).

### Measurements

#### 24-hour movement behaviours and sleep

Detailed procedures for accelerometer data collection and processing have been published previously [42]. In brief, at the end of the clinical assessment, participants were asked to wear two AX3 accelerometers (Axivity, Newcastle, UK) for seven days, one on the right thigh and one on the lower back. After completion, the accelerometers were returned either in a pre-stamped envelope or in person at the field station. The accelerometer data was sampled at 50 Hz with an 8G bandwidth and stored on the AX3 internal flash drive. The OmGui software (v 1.0.0.43; Open movement, Newcastle, UK) was used to configure, initialise, and download the data. The downloaded data streams from the two accelerometers were synchronised and segmented into 5-s windows.

All machine learning model development and validation procedures are described in detail elsewhere [26, 38, 39]. First, a machine learning model was used to classify non-wear time with high overall accuracy (97%), and if non-wear was predicted for at least one hour, the entire 24-h period for that day was excluded from further analyses [43]. Second, two independent machine learning models were used to predict 1) key daily physical activities (standing, walking, running, cycling) and postures (sitting and lying) and 2) sleep/wake for days with valid accelerometer data (midnight-to-midnight) [26, 38, 39]. Key physical activities and postures were validated in adults (*N* = 22, 25–68 years) equipped with a chest-mounted camera following a semi-structured free-living protocol showing high overall accuracy (96%) [26, 38], and later repeated in older adults (*N* = 18, 70–95 years) showing a slightly lower overall accuracy (91%) [44]. Sleep/wake was validated in adults (*N* = 29, 17–70 years) using overnight polysomnography as ground truth, showing high overall accuracy (84%), excellent sensitivity (0.95) and moderate specificity (0.72) [39].

During post-processing of accelerometer data, time classified as sleep was subtracted from time classified as lying, leaving residual lying time classified as “lying awake”. Time spent standing, walking, running, cycling, sitting, lying awake, and sleeping was averaged across days with valid data. Mean (SD) and median (IQR) minutes spent running per 24 h were 0.2 (1.6) and 0.0 (0.01), respectively. The corresponding figures for cycling were 4.6 (7.2) and 2.2 (4.2), respectively. Accordingly, running and cycling accounted for approximately 0.01–0.03% of the 24-h day and were therefore omitted from further analyses.

#### Cognitive status

MCI and dementia diagnostic procedures in HUNT4 70+ have been detailed elsewhere [36, 37]. Briefly, trained health personnel assessed cognitive, functional, and neuropsychiatric status at designated field stations or the participant’s primary residence, using standardised tests, participant and proxy interviews, and clinical assessments. Two scientific and clinical experts, from a panel of nine, independently reviewed all available information for each participant to determine whether the individual met criteria for MCI or dementia, and to assess dementia severity according to the Diagnostic and Statistical Manual of Mental Disorders, 5th edition (DSM-5) [45] based on a consensus approach. If consensus was not reached, a third expert was consulted. Those with dementia were further classified as having mild, moderate, or severe dementia and as Alzheimer’s dementia (AD), vascular dementia (VaD), Lewy body dementia (LBD, including dementia with Lewy bodies and Parkinson’s disease dementia), frontotemporal dementia (FTD), mixed dementia, or unspecified dementia, based on DSM-5 criteria [45]. Before the consensus diagnostic procedure, all nine experts independently assessed a set of 50 calibration cases. Overall inter-rater reliability was substantial (Fleiss’ kappa 0.70, 95% CI 0.66, 0.74), and excellent for dementia (0.90, 95% CI 0.79, 0.90) [36]. All-cause dementia was included in the current study. Participants not diagnosed with MCI or dementia were categorised as cognitively unimpaired. Function in activities of daily life and global cognition in the study sample are reported in Additional file 1, Table A1.

#### Sample characteristics and potential confounders

The relationship between movement behaviours, sleep, cognition, and brain health is influenced by a wide range of individual, social, and environmental factors [46–48]. To minimise confounding while avoiding collinearity and over-adjustment of estimates, based on current knowledge and available HUNT data, we included age, sex, marital status, education, self-rated health, body mass index (BMI), depression, alcohol consumption, and smoking status as sample characteristics and potential confounders.

Age, sex, education, and marital status (“married”, “unmarried”, “divorced”, and “widowed”) were obtained from Statistics Norway and linked to each participant using the unique personal identification number assigned to all Norwegian residents. Education was recorded according to the International Standard Classification of Education 2011, with the highest level achieved classified as primary (≤ 10 years), secondary (11–13 years), and higher (≥ 14 years). Self-related health was assessed using the questionnaire item “How is your current health?” with response options “very good”, “good”, “not so good”, and “poor” [49]. Depression was assessed using the Hospital Anxiety and Depression scale, depression subscale (HADS-D), which contains seven questions and yields a 0–21 sum score, with a higher score indicating higher levels of depression [50]. The HADS-D is validated in older adults [51], and based on a previous validation study, it was dichotomised into no depression (< 8) or depressed (≥ 8) [52]. Alcohol consumption was assessed using the questionnaire item “How often during the last 12 months did you drink alcohol?” with response options “never had alcohol”, “not at all the last year”, “ ≤ 1 time/month”, “2–4 times/month”, “2–3 times/week”, “ ≥ times/week”, and was categorised as “abstainer/former”, “occasional”, and “regular/frequent” [53]. Smoking was self-reported as “never”, “previous”, and “current” (including “sometimes” and “daily”). Weight and height were measured using a digital flat scale (Seca 813, Germany) and a stadiometer (Seca 217, Germany). BMI was calculated as weight (kg) divided by height squared (m^2^); obesity was defined as a BMI ≥ 30 kg/m^2^.

### Statistical analyses

All analyses were conducted using STATA 19.0 BE (StataCorp LLC, College Station, TX, USA). Participants included in the study sample (*n* = 4,699) were more likely to be younger, married, higher educated, assessed at field stations, to report better self-rated health, and to be cognitively unimpaired than eligible participants not included (*n* = 4,463) (all *p* < 0.05). To address this potential selection bias, inverse probability weights (IPWs) were constructed using an indicator variable for participation. The indicator variable for participation was used as the dependent variable. Based on a sensitivity analysis of eligible participants with and without accelerometer data, four logistic regression models were tested, and the model with the lowest Akaike Information Criterion (AIC) was selected. Predictors in the final model included age (classified into 5-year groups), sex, marital status, education, test location (field station/ primary residence), self-rated health, and cognitive status. Weights were truncated at the 1st (1.41, *n* = 75) and 99th (4.28, *n* = 84) percentiles to reduce the influence of extreme values (*n* = 159, 1.7%). Mean IPW by age group, sex, education, and cognitive status is provided in Additional file 1, Table A2.

Descriptive statistics were used to summarise sample characteristics and potential confounders, stratified by cognitive status (i.e., cognitively unimpaired, MCI, and dementia). Survey-weighted descriptive statistics were used to summarise the average time spent standing, walking, sitting, lying awake, and sleeping across the three groups.

Among the sample characteristics and potential confounders, five variables had missing data: self-rated health (*n* = 178), BMI (*n* = 251), depression (*n* = 505), alcohol consumption (*n* = 178), and smoking (*n* = 116). In total, 82% had complete data. The proportion of missing data was highest among people with dementia (Additional file 1, Table A3). To manage missing data, we used multiple imputation by chained equations (MICE). Variables without missing data served as predictors. We checked for multicollinearity among partially observed variables, predictors, and combinations of both. Twenty datasets were imputed, each with 50 iterations per chain. Results from the imputed datasets were combined using Rubin’s rules to ensure valid statistical inferences. Summary statistics for imputed variables were consistent with crude estimates.

To quantify differences in time accumulated in movement behaviours and sleep across cognitive groups, they were analysed separately using survey-weighted multiple linear regression analyses. The dependent variables were standing, walking, sitting, lying awake, and sleeping, and the independent variable was cognitive status (i.e., cognitively unimpaired, MCI, dementia). In model 1, we adjusted for age, sex, and education. In model 2, we further adjusted for marital status, self-rated health, obesity, depression, alcohol consumption, and smoking. The cognitively unimpaired group served as the reference for comparisons with participants with MCI and dementia. The MCI group served as the reference for comparisons with participants with dementia.

## Results

### Participant characteristics

Table 1 presents sample characteristics for all participants by cognitive status. Across all groups, mean age was 75.9 years (SD 4.8, range 70–100), and about half were women. Cognitively unimpaired participants were more likely to be younger, married, higher educated, and to report better health than those with MCI and dementia. Among participants with dementia, most had mild dementia (74.8%), followed by moderate (23.6%) and severe dementia (1.9%). This distribution was similar among eligible participants with dementia who did not provide valid accelerometer data (*n* = 648) (*p* > 0.05).

**Table 1:** Sample characteristics by cognitive status

Variables	Cognitively unimpaired	MCI	Dementia
	(*n* = 2,649)	(*n* = 1,690)	(*n* = 360)
Age, years, mean (SD)	75.9 (4.8)	76.7 (5.5)	81.4 (7.2)
Sex, women, *n* (%)	1,456 (55.0)	854 (50.5)	184 (51.1)
Marital status, *n* (%)			
Married	1,793 (69.7)	1,079 (63.8)	177 (49.2)
Unmarried	84 (3.2)	69 (4.1)	17 (4.7)
Divorced	257 (9.7)	171 (10.1)	40 (11.1)
Widowed	515 (19.4)	371 (22.0)	126 (35.0)
Education, *n* (%)			
< 10 years	421 (15.9)	408 (24.1)	136 (37.8)
10–13 years	1,436 (54.2)	959 (56.8)	174 (48.3)
≥ 14 years	792 (29.9)	323 (19.1)	50 (13.9)
Self-rated health, *n* (%)			
Poor	29 (1.1)	16 (1.0)	20 (6.5)
Not so good	643 (24.9)	568 (34.7)	150 (48.9)
Good	1,648 (63.9)	944 (57.7)	128 (41.7)
Very good	258 (10.0)	108 (6.6)	9 (2.9)
BMI, kg/m^2^, mean (SD)	27.0 (4.1)	27.2 (4.2)	26.3 (4.3)
Obesity, BMI ≥ 30, *n* (%)	512 (19.6)	367 (22.9)	40 (17.4)
* Depression, yes, *n* (%)	183 (7.6)	158 (10.5)	62 (22.6)
Alcohol, *n* (%)			
Abstainer/former	397 (15.3)	311 (19.1)	103 (34.0)
Occasional	1,509 (58.2)	1,003 (61.7)	155 (51.2)
Regular/frequent	685 (26.4)	313 (19.2)	45 (14.8)
Smoking, *n* (%)			
Never	1,065 (40.7)	605 (36.5)	117 (37.6)
Previous	1,395 (53.4)	950 (57.3)	171 (55.0)
Current	154 (5.9)	103 (6.2)	23 (7.4)
Accelerometer wear time, days, mean (SD)	5.9 (0.8)	5.8 (0.9)	5.8 (0.9)
Dementia severity, *n* (%)			
Mild			268 (74.4)
Moderate			85 (23.6)
Severe			7 (1.9)
Dementia diagnosis, *n* (%)			
AD			208 (57.8)
VaD			42 (11.7)
LBD			8 (2.2)
FTD			8 (2.2)
Mixed			36 (10.0)
Unspecified			58 (16.1)

*MCI* Mild cognitive impairment, *BMI* Body mass index, *AD* Alzheimer’s dementia, *VaD* Vascular dementia, *LBD* Lewy Body dementias, *FTD* Frontotemporal dementia

^*^Depression was assessed with the Hospital Anxiety Depression scale, Depression subscale (HADS-D) and defined as a sum score of ≥ 8 (range 0–21)

### Distribution of 24-hour movement behaviours and sleep

**Fig. 2:**
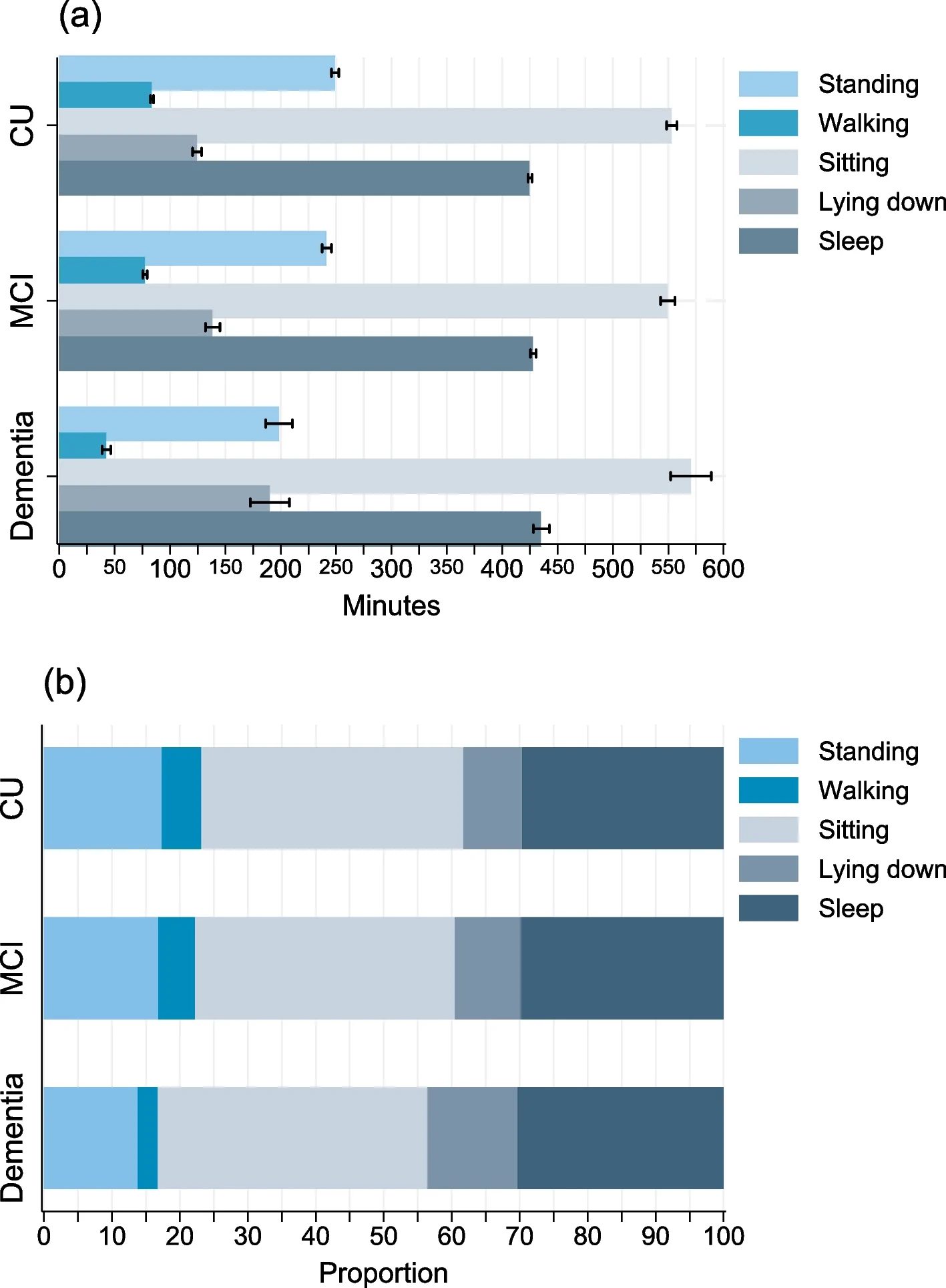
The (**a**) absolute (with 95% CI error bars) and (**b**) relative contribution of standing, walking, sitting, lying awake, and sleep to the 24-h day. CU = cognitively unimpaired; MCI = mild cognitive impairment. Estimates are based on included participants and survey-weighted to account for eligible HUNT4 70 + participants without valid accelerometer data

The distribution of 24-h movement behaviours and sleep is shown in Fig. 2 and Additional file 1, Table A4. Cognitively unimpaired participants spent 17.4% of the day standing (4.2 h), 5.8% walking (83.7 min), 38.5% sitting (9.2 h), 8.7% lying awake (2.1 h), and 29.6% sleeping (7.1 h). Participants with MCI spent 16.8% of the day standing (4.0 h), 5.4% walking (77.3 min), 38.3% sitting (9.2 h), 9.7% lying awake (2.3 h), and 29.8% sleeping (7.1 h). The corresponding values for participants with dementia were 13.8% (3.3 h) standing, 3.0% (42.5 min) walking, 39.7% (9.5 h) sitting, 13.2% (3.2 h) lying awake, and 30.3% (7.3 h) sleeping.

### Differences in 24-hour movement behaviours and sleep

**Fig. 3:**
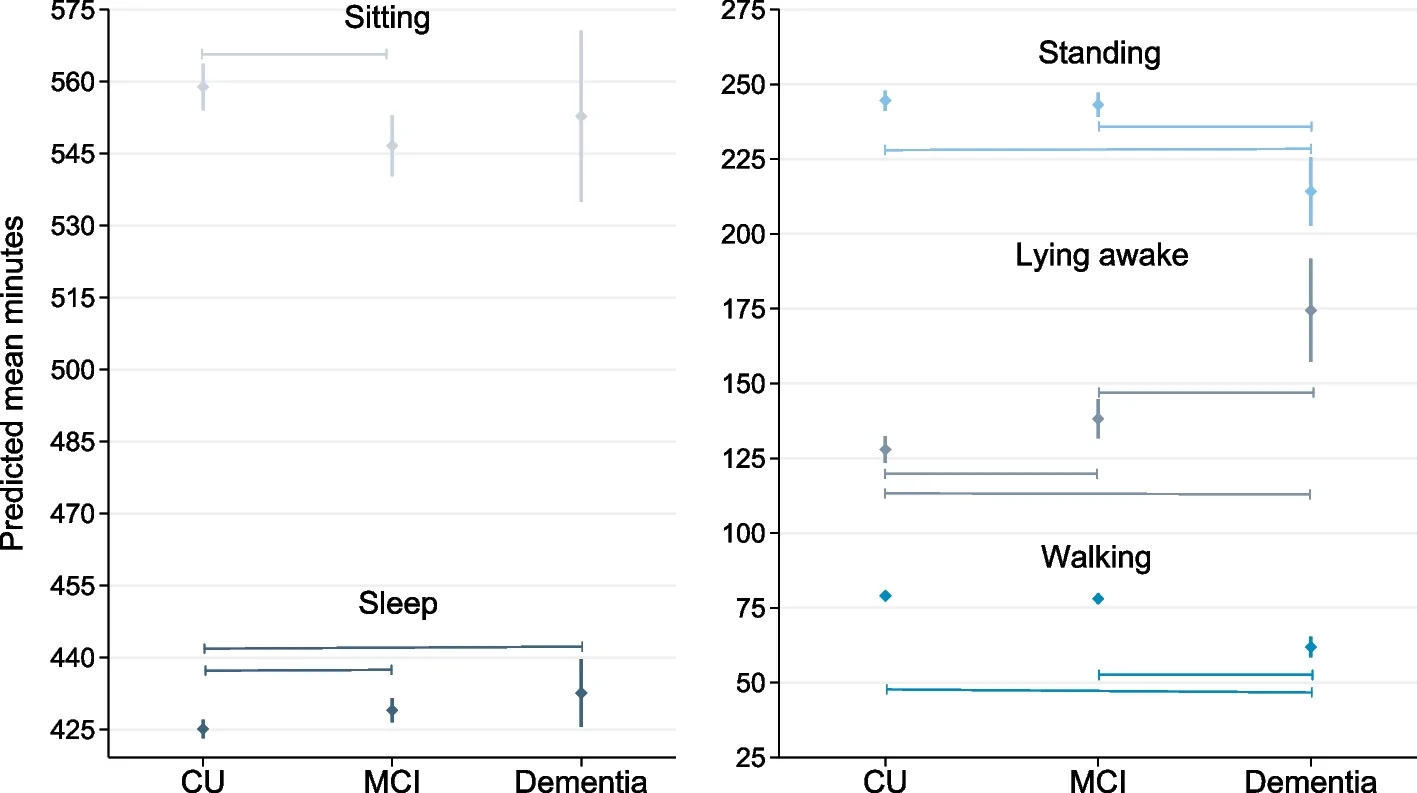
Margins plot of adjusted means (with 95% CI). CU = cognitively unimpaired; MCI = mild cognitive impairment. Adjusted for age, sex, education, marital status, self-rated health, obesity, depression, alcohol consumption, and smoking. Horizontal lines indicate significant differences between groups. Estimates are based on included participants and survey-weighted to account for HUNT4 70 + participants without valid accelerometer data

Time spent in key daily physical activities, postures, and sleep across the cognitive spectrum is shown in Table 2 and Fig. 3. In fully adjusted models, participants with MCI spent less time sitting (β = −12.4 min, 95% CI: −20.0, −4.4) and more time lying awake (β = 10.3 min, 95% CI: 2.7, 17.9) and sleeping (β = 3.9 min, 95% CI: 0.8, 7.0) per 24-h than cognitively unimpaired participants. Standing time did not differ significantly, whereas walking time differed only in partially adjusted models (β = −4.5 min, 95% CI: −9.9, −2.1).

**Table 2:** Survey-weighted multiple linear regression of time spent in key daily physical activities, postures and sleep per 24-hours across the cognitive spectrum

Variables	Ref. Cognitively unimpaired				Ref. MCI	
	**MCI**		**Dementia**		**Dementia**	
	**ß (95% CI)**	***p***	**ß (95% CI)**	***p***	**ß (95% CI)**	***p***
*Standing, min	−3.5 (−8.7, 1.7)	.2	−43.6 (−56.0, −32.2)	≤.001	−40.1 (−52.7, −27.5)	≤.001
*Walking, min	−4.5 (−6.9, −2.1)	≤.001	−35.1 (−39.3, −31.0)	≤.001	−30.6 (−34.9, −26.4)	≤.001
*Sitting, min	−8.7 (−16.7, −0.7)	.03	7.6 (−11.3, 26.5)	.4	16.3 (−3.2, 35.8)	.1
*Lying awake, min	12.7 (5.0, 20.3)	.001	61.8 (44.4, 79.3)	≤.001	49.2 (30.8, 67.6)	≤.001
*Sleep, min	3.8 (0.7, 6.9)	.01	10.7 (3.3, 18.1)	.005	6.9 (−0.7, 14.5)	.07
†Standing, min	−1.4 (−6.5, 3.8)	.6	−30.3 (−42.5, −18.2)	≤.001	−29.0 (−41.3, −16.5)	≤.001
†Walking, min	−1.0 (−3.1, 1.1)	.3	−17.2 (−20.9, −13.4)	≤.001	−16.1 (−19.9, −12.4)	≤.001
†Sitting, min	−12.4 (−20.0, −4.4)	.002	−6.1 (−24.9, 12.7)	.5	6.3 (−13.3, 25.6)	.5
†Lying awake, min	10.3 (2.7, 17.9)	.008	46.5 (28.7, 64.5)	≤.001	36.3 (17.1, 55.4)	≤.001
†Sleep, min	3.9 (0.8, 7.0)	.01	7.5 (0.9, 14.9)	.04	3.6 (−4.0, 11.2)	.3

Estimates are based on included participants and survey-weighted to account for eligible HUNT4 70 + participants without valid accelerometer data

*MCI* Mild cognitive impairment

^*^Adjusted for age, sex, and education

^†^Adjusted for age, sex, education, marital status, self-rated health, obesity (body mass index ≥ 30), depression (Hospital Anxiety and Depression scale, Depression subscale ≥ 8), alcohol consumption, and smoking

*p* = survey-weighted linear regression

Participants with dementia spent 29.0 and 30.3 (95% CI: −41.3, −16.5 and −42.5, −18.2) minutes less standing, 16.1 and 17.2 (95% CI: −19.9, −12.4 and −20.9, −13.4) minutes less walking, and 36.3 and 46.5 (95% CI: 17.1, 55.4 and 28.7, 64.5) minutes more lying awake per 24-h than participants with MCI and those who were cognitively unimpaired, respectively. Additionally, participants with dementia spent 7.5 (95% CI: 0.9, 14.9) minutes more sleeping per 24-h than cognitively unimpaired participants. Similar distributions were observed, with slightly larger effect sizes in partially adjusted models.

## Discussion

The aim of this population-based study was to describe and compare the distribution of 24-h movement behaviours and sleep across the cognitive spectrum in older adults. Overall, older adults across the cognitive spectrum spent most of the day sitting and sleeping, followed by standing, lying awake, and walking. However, they exhibited distinct distributions of 24-h movement behaviours and sleep. Older adults with MCI spent less time sitting and more time lying awake and sleeping than their cognitively unimpaired peers, while time spent standing and walking was similar. Older adults with dementia spent less time standing and walking, and more time lying awake, than those with MCI and cognitively unimpaired peers, and more time sleeping than their cognitively unimpaired peers.

We did not observe differences in time spent standing or walking between participants with MCI and cognitively unimpaired older adults. Methodological heterogeneity across studies, including MCI classification and procedures for device-based measurement, complicates direct comparisons, but supports previous findings that there are no differences in PA levels in people with MCI compared to cognitively unimpaired individuals [19]. Compared with a prior 24-h day study of PA, SB, and sleep in MCI [54], MCI participants in the current study spent less overall time in PA. Preserved independent function in activities of daily life is embedded in the MCI diagnostic criteria, but previous studies have demonstrated the presence of functional impairments including slow walking speed, reduced muscle strength, and reduced balance in MCI [55, 56]. The similar walking and standing times observed between older adults with MCI and cognitively unimpaired peers may suggest that overall engagement in these behaviours is preserved.

Older adults with dementia spent substantially less time standing and walking than their cognitively unimpaired peers. These findings are consistent with previous studies on reduced PA [14–16] and on standing and walking in dementia [14], strengthening the current evidence base with a large, population-based sample and standardised diagnostic criteria. Our findings further extend earlier work showing less time spent standing and walking between people with dementia and those with MCI. Dependence in activities of daily life is part of the diagnostic distinction between MCI and dementia, and reduced time spent standing and walking in dementia may reflect functional impairments embedded in the clinical manifestation of the disease. Further, insufficient PA is a risk factor for dementia [4, 5, 9], and less time spent standing and walking may reflect long-term behavioural patterns. Standing and walking are fundamental to mobility and participation and play a central role in everyday life. Given international guidelines that all movement, regardless of intensity, matters for health [57], the observed PA deficits in dementia underscore the importance of monitoring and implementing interventions to increase PA in people with dementia.

Across all groups, sitting and lying awake together comprised nearly half of the 24-h day, consistent with previous research on SB in older adults [33, 58]. Although total sedentary time was similar across groups, the contribution of sitting versus lying awake differed. Older adults with MCI spent less time sitting but more time lying awake than their cognitively unimpaired peers. Those with dementia spent a similar amount of time sitting to those with MCI and cognitively unimpaired peers, but substantially more time lying awake. While sitting and lying awake involve similar energy expenditure [28], these movement behaviours differ functionally and contextually, with sitting often associated with greater social and cognitive activity [31, 33]. Differences in the composition of SB may suggest that sitting and lying awake may have different functional and behavioural implications for cognitive health and warrant further research. They may, however, also reflect impairments embedded in the clinical manifestation of the disease. In dementia, poor sleep quality, fragmentation, and reduced deep sleep are common and contribute to daytime sleepiness [17]. These dementia symptoms, combined with behavioural and psychiatric symptoms such as apathy and anxiety and functional limitations, may have contributed to higher lying-awake time in this group. Without additional contextual information, the reasons for the observed differences in sitting and lying awake remain uncertain.

Sleep accounted for approximately one-third of the 24-h day across all groups, and, on average, older adults met the recommended sleep duration (7–8 h) [59]. However, older adults with MCI and dementia spent slightly more time sleeping (< 10 min per 24-h) than cognitively unimpaired peers. Longer sleep duration in MCI and dementia is consistent with previous findings [17, 23], but the clinical relevance of the findings observed in this study remains uncertain. Although accelerometry allowed differentiation between sleep and wakefulness, other key features of sleep, such as quality, fragmentation, and timing, were not assessed. The importance of total sleep duration and the interrelationships among sitting, lying awake, and sleep remain uncertain and warrant further investigation.

Key strengths of this study include a large population-based sample, comprehensive cognitive assessments, and device-measured movement behaviours derived from validated machine-learning models. Still, the study has several limitations. Fully adjusted models showed several statistically significant differences in time spent in different behaviours between groups, but the clinical importance of the effect sizes remains unknown. Additionally, movement behaviours and sleep were analysed separately, but are interdependent, comprising a finite 24-h day. In our descriptive approach, we did not account for their compositional structure, and findings should be interpreted as descriptive differences in accumulated time. Future studies applying compositional data analyses should be conducted to provide insights into how reallocating time between behaviours is associated with cognitive status. Further, data on movement behaviour and sleep temporal patterns (bouts, sequencing, timing, fragmentation) were not available, thereby precluding any such inferences. The relationship between movement behaviours, sleep, and cognitive impairment is influenced by a wide range of individual, social, and environmental factors. The study accounted for several important factors, but residual confounding from unmeasured factors such as comorbidities, medications, pain, and use of assistive devices might have influenced estimates and remain a limitation. Further, the study’s cross-sectional design precludes causal inference. A substantial proportion of eligible participants lacked accelerometer data, and the reasons for missing data were not specified in the dataset (e.g., unwillingness to wear an accelerometer, lack of availability during the clinical examination, malfunction or technical issues, or invalid accelerometer data). To mitigate concerns about selection bias, IPW was applied, and sensitivity analyses showed no differences in dementia severity between participants with and without accelerometer data. Despite this, residual selection bias cannot be excluded, and the absence of information regarding missing accelerometer data remains a limitation. Despite the high overall accuracy of the machine-learning models, misclassification may still have occurred, particularly when distinguishing between standing and walking in older adults [44]. Finally, movement behaviour and sleep models have not been validated in persons with MCI or dementia. In these groups, classification accuracy may be reduced, misclassification cannot be excluded and should be considered when interpreting results.

## Conclusion

Older adults across the cognitive spectrum exhibit distinct distributions of 24-h movement behaviours and sleep. MCI is characterised by less sitting and more time spent lying awake and sleeping, alongside preserved standing and walking, compared with cognitively unimpaired peers. Dementia is characterised by less standing and walking, more time lying awake compared with MCI and cognitively unimpaired peers, and more time sleeping than cognitively unimpaired peers. The clinical importance of the observed differences remains unknown, and future research on the independent and compositional effects of these behaviours is warranted.

## Supplementary Information

Additional file 1. Table A1. Mean inverse probability weights by age group, sex, education, and cognitive status. Table A2. Count and proportion of missing by cognitive status. Table A3. Average time spent standing, walking, sitting, lying awake, and sleeping per 24-hours.

Additional file 2. STROBE Checklist: Cross-sectional studies.

## Data Availability

The data supporting the findings of this study are available from the HUNT Research Centre (https://www.ntnu.edu/hunt), but restrictions apply to these data, which were used under license for the current study. However, data are available from co-author “Paul Jarle Mork” (Email ID: paul.mork@ntnu.no), upon reasonable request and with permission from the HUNT Research Centre (https://www.ntnu.edu/hunt).

## Abbreviations

MCI: Mild cognitive impairment
PA: Physical activity
SB: Sedentary behaviour
HUNT4 70 +: The Trøndelag Health Study, 4th survey, 70-years-and-older cohort
BMI: Body mass index
HADS-D: Hospital Anxiety and Depression scale, depression subscale
DSM-5: Diagnostic and Statistical Manual of mental disorders, 5th
AD: Alzheimer’s dementia
VaD: Vascular dementia
LBD: Lewy body dementia
FTD: Frontotemporal dementia
IPW: Inverse probability weights
MICE: Multiple imputation by chained equations
