# Supplementary material for: Distribution of device-measured 24-hour movement behaviours across the cognitive spectrum in older adults: the HUNT study
Source: Eur Rev Aging Phys Act. 2026 Aug 13;23:40. doi: 10.1186/s11556-026-00433-x (PMC13753409; doi:10.1186/s11556-026-00433-x)
Supplement: Supplementary file 1 [file 11556_2026_433_MOESM1_ESM.docx]

# **Additional file 1**

**Distribution of device-measured 24-hour movement behaviours across the cognitive spectrum in older adults: the HUNT study**

Table of contents

**Table A1.** Function in activities of daily life and global cognition in the study sample..… **p. 2**

**Table A2.** Mean inverse probability weights by age group, sex, education, and cognitive status………………………………………………………………………………………. **p. 3**

**Table A3.** Count and proportion of missing by cognitive status………………………….. **p. 4**

**Table A4.** Average time spent standing, walking, sitting, lying down (awake), and sleeping per 24-hours……………………………………………………………………………….. **p. 4**

**Table A1.** Function in activities of daily life and global cognition in the study sample

| **Variables** | **Cognitively unimpaired** | **MCI** | **Dementia** |
| --- | --- | --- | --- |
|  | (*n* = 2,649) | (*n* = 1,690) | (*n* = 360) |
| *6 item pADL, n (%) |  |  |  |
| Need help in all pADL | 24 (1.0) | 27 (1.7) | 5 (1.7) |
| Need help in 1–6 pADL | 38 (1.5) | 39 (2.5) | 80 (26.6) |
| Independent in pADL | 2,394 (97.5) | 1,496 (95.8) | 216 (71.8) |
| *missing* | 193 (7.3) | 128 (7.6) | 59 (16.4) |
| *5 item iADL, n (%) |  |  |  |
| Need help in all iADL | 0 (0) | 6 (0.4) | 36 (12.1) |
| Need help in 1–5 iADL | 285 (11.6) | 314 (20.3) | 127 (42.8) |
| Independent in iADL | 2,164 (88.4) | 1,230 (79.3) | 134 (45.1) |
| *missing* | 200 (7.6) | 140 (8.3) | 63 (17.5) |
| **MoCA, mean (sd) | 25.6 (2.3) | 21.9 (3.0) | 15.1 (4.4) |
| *missing* | 57 (2.1) | 66 (3.9) | 17 (4.7) |
| MCI = mild cognitive impairment; pADL = personal activities of daily life; iADL instrumental activities of daily life; MoCA = Montreal Cognitive Assessment.  *Grov EK, Fosså SD, Dahl AA. Activity of daily living problems in older cancer survivors: a population-based controlled study. *Health Soc Care Community*. 2010;18(4):396-406. doi:10.1111/j.1365-2524.2010.00912.x  ** Nasreddine ZS, Phillips NA, Bédirian V, et al. The Montreal Cognitive Assessment, MoCA: a brief screening tool for mild cognitive impairment. *J Am Geriatr Soc*. 2005;53(4):695-699. doi:10.1111/j.1532-5415.2005.53221.x | | | |

**Table A2.** Mean inverse probability weights by age group, sex, education, and cognitive status

|  |  |  | **Mean weights** | | |
| --- | --- | --- | --- | --- | --- |
| **Age group** | **Sex** | **Education** | **Cognitively unimpaired** | **MCI** | **Dementia** |
| 70-74 | Women | <10 years | 1.95 | 2.06 | 2.57 |
|  |  | 10–12 years | 1.79 | 1.86 | 2.24 |
|  |  | ≥13 years | 1.59 | 1.66 | 1.95 |
|  | Men | <10 years | 1.97 | 2.12 | 2.72 |
|  |  | 10–12 years | 1.81 | 1.90 | 2.32 |
|  |  | ≥13 years | 1.61 | 1.69 | 2.01 |
| 75-79 | Women | <10 years | 1.98 | 2.07 | 2.63 |
|  |  | 10–12 years | 1.78 | 1.86 | 2.31 |
|  |  | ≥13 years | 1.61 | 1.67 | 2.08 |
|  | Men | <10 years | 1.96 | 2.10 | 2.74 |
|  |  | 10–12 years | 1.79 | 1.89 | 2.37 |
|  |  | ≥13 years | 1.60 | 1.68 | 2.05 |
| 80-84 | Women | <10 years | 2.24 | 2.42 | 3.13 |
|  |  | 10–12 years | 2.03 | 2.17 | 2.82 |
|  |  | ≥13 years | 1.80 | 1.85 | 2.34 |
|  | Men | <10 years | 2.26 | 2.37 | 3.05 |
|  |  | 10–12 years | 2.03 | 2.11 | 2.78 |
|  |  | ≥13 years | 1.76 | 1.82 | 2.32 |
| 85-89 | Women | <10 years | 2.72 | 2.96 | 3.82 |
|  |  | 10–12 years | 2.39 | 2.67 | 3.53 |
|  |  | ≥13 years | 2.09 | 2.20 | 3.01 |
|  | Men | <10 years | 2.65 | 2.76 | 3.99 |
|  |  | 10–12 years | 2.32 | 2.51 | 3.32 |
|  |  | ≥13 years | 2.06 | 2.29 | 3.25 |
| ≥90 | Women | <10 years | 2.97 | 3.07 | 3.88 |
|  |  | 10–12 years | 2.51 | 2.70 | 2.53 |
|  |  | ≥13 years | 2.68 | 2.55 | 2.64 |
|  | Men | <10 years | 2.70 | 3.18 | 3.64 |
|  |  | 10–12 years | 2.44 | 2.59 | 3.45 |
|  |  | ≥13 years | 2.19 | 2.13 | 3.62 |
| MCI = mild cognitive impairment.  E.g. for women 70-74 years old with less than 10 years education the mean predicted value of participation was 0.512, 0.485, 0.389 for CU, MCI and dementia participants, giving mean weights 1.95 (1/0.512), 2.06 (1/0.485), and 2.57 (1/0.389). For men 70-74 years old with less than 10 years education the mean predicted value of participation was 0.507, 0.472, 0.368 for CU, MCI and dementia participants, giving mean weights 1.97, 2.12 and 2.72. | | | | | |

**Table A3.** Count and proportion of missing by cognitive group

|  | **Cognitively unimpaired** | **MCI** | **Dementia** |
| --- | --- | --- | --- |
| **Variable** | (*n* = 2,649) | (*n* = 1,690) | (*n* = 360) |
| Self-rated health, n (%) | 71 (2.7) | 54 (3.2) | 53 (14.7) |
| BMI, n (%) | 36 (1.4) | 85 (5.0) | 130 (36.1) |
| *Depression, n (%) | 236 (8.9) | 183 (10.8) | 86 (23.9) |
| Alcohol consumption, n (%) | 58 (2.2) | 63 (3.7) | 57 (15.8) |
| Smoking, n (%) | 35 (1.3) | 32 (1.9) | 49 (13.6) |
| MCI = mild cognitive impairment; BMI = body mass index  * Depression was assessed with the Hospital Anxiety Depression scale, Depression subscale (HADS-D) and depression was categorized as sum ≥8. | | | |

**Table A4.** Average time spent standing, walking, sitting, lying down (awake), and sleeping per 24-hours

| **Variable** | **Cognitively unimpaired** | **MCI** | **Dementia** | |
| --- | --- | --- | --- | --- |
|  | Mean (SD) | Mean (SD) | Mean (SD) | |
| Standing, min | 249.1 (86.5) | 241.5 (84.4) | 198.5 (90.7) | |
| Walking, min | 83.7 (39.0) | 77.3 (38.9) | 42.5 (31.6) | |
| Sitting, min | 553.1 (124.3) | 549.5 (131.5) | 570.6 (137.7) | |
| Lying awake, min | 124.3 (107.4) | 138.6 (128.7) | 190.0 (131.4) | |
| Sleep, min | 425.1 (48.1) | 428.2 (51.1) | 435.4 (52.6) | |
| MCI = mild cognitive impairment.  Estimates are based on included participants and survey-weighted to account for eligible HUNT4 70+ participants without valid accelerometer data. | | | |  |
